# Plastid Genomes of Five Species of Riverweeds (Podostemaceae): Structural Organization and Comparative Analysis in Malpighiales

**DOI:** 10.3389/fpls.2019.01035

**Published:** 2019-08-20

**Authors:** Ana M. Bedoya, Bradley R. Ruhfel, C. Thomas Philbrick, Santiago Madriñán, Claudia P. Bove, Attila Mesterházy, Richard G. Olmstead

**Affiliations:** ^1^Department of Biology and Burke Museum, University of Washington, Seattle, WA, United States; ^2^University of Michigan Herbarium, University of Michigan, Ann Arbor, MI, United States; ^3^Department of Biological and Environmental Sciences, Western Connecticut State University, Danbury, CT, United States; ^4^Laboratorio de Botánica y Sistemática, Departamento de Ciencias Biológicas, Universidad de los Andes, Bogotá, Colombia; ^5^Departamento de Botânica, Museu Nacional, Universidade Federal do Rio de Janeiro, Rio de Janeiro, Brazil; ^6^Directorate of Hortobágy National Park, Debrecen, Hungary

**Keywords:** genome rearrangements, Malpighiales, phylogenomics, plastome, Podostemaceae

## Abstract

With the advent of next-generation sequencing technologies, whole-plastome data can be obtained as a byproduct of low-coverage sequencing of the plant genomic DNA. This provides an opportunity to study plastid evolution across groups, as well as testing phylogenetic relationships among taxa. Within the order Malpighiales (∼16,000 spp.), the Podostemaceae (∼300 spp.) stand out for their unique habit, living attached to rocks in fast-flowing aquatic habitats, and displaying highly modified morphologies that confound our understanding of their classification, biology, and evolution. In this study, we used genome skimming data to assemble the full plastid genome of 5 species within Podostemaceae. We analyzed our data in a comparative framework within Malpighiales to determine the structure, gene content, and rearrangements in the plastomes of the family. The Podostemaceae have one of the smallest plastid genomes reported so far for the Malpighiales, possibly due to variation in length of inverted repeat (IR) regions, gene loss, and intergenic region variation. We also detected a major inversion in the large single-copy region unique to the family. The uncommon loss or pseudogenization of *ycf1* and *ycf2* in angiosperms and in land plants in general is also found to be characteristic of Podostemaceae, but the compensatory mechanisms and implications of this and of the pseudogenization of *accD* and *rpl23* and loss of *rps16* remain to be explained in this group. In addition, we estimated a phylogenetic tree among selected species in Malpighiales. Our findings indicate that the Podostemaceae are a distinct lineage with long branches that suggest faster rates of evolution in the plastome of the group, compared with other taxa in the order. This study lays the foundations for future phylogenomic studies in the family.

## Introduction

The plastids have a relatively small, maternally inherited, haploid genome (Sugiura, 1992). It ranges between 120 and 170 kb in length and is generally composed of a circular structure with two IRs that are mirror images in terms of gene content (IRa and IRb), separated from each other by a large and a small single-copy regions (LSC and SSC, respectively) (Downie and Palmer, 1992; Sugiura, 1992). Because the plastome encodes genes that are essential for fundamental processes such as photosynthesis and its own replication, it has been generally understood that its genome shows a relatively high degree of conservation in size, structure, and gene content within land plants (Palmer, 1985; Wicke et al., 2011). However, structural rearrangements, gene losses, and expansions and contractions in IRs are widely documented across species (Goulding et al., 1996; Krause, 2011; Weng et al., 2014; Schwarz et al., 2015; Xu et al., 2015; Rabah et al., 2019; Shrestha et al., 2019). Such rearrangements have been relevant in a systematic framework when supporting the monophyly of certain groups (Jansen and Palmer, 1987; Downie and Palmer, 1992; Hoot and Palmer, 1994; Cosner et al., 2004).

With the advent of next-generation sequencing technologies, information from whole-genome data is quickly available at a low cost (Metzker, 2009). Given that plastomes exist in high copy numbers in plant cells, even a genome skimming approach where the nuclear genome is sequenced at low-coverage provides a mechanism to obtain a fully assembled plastome as a byproduct (Straub et al., 2012; Olmstead and Bedoya, 2019). Over the past few years, this has provided the advantage of rapidly generating whole-plastid sequences for a large number of taxa (Daniell et al., 2016). This information has been used to disentangle phylogenetic relationships and to study plastid evolution in selected groups of plants (Ruhfel et al., 2014; Cauz-Santos et al., 2017; Firetti et al., 2017; Gitzendanner et al., 2018; Li and Zheng, 2018; Liu et al., 2018; Li et al., 2019; Lloyd Evans et al., 2019).

Malpighiales is a large order with 36 families, more than 700 genera, and ∼16,000 species (Wurdack and Davis, 2009; The Angiosperm Phylogeny Group, 2016). Full plastid assemblies for 111 species in the families Chrysobalanaceae, Clusiaceae, Erythroxylaceae, Euphorbiaceae, Linaceae, Malpighiacee, Passifloraceae, Salicaceae, and Violaceae currently reside in the NCBI database. In addition, previous studies using whole-plastome data of *Passiflora edulis* Sims (Cauz-Santos et al., 2017) and of *Byrsonima crassifolia* (L.) Kunth and *Byrsonima coccolobifolia* Kunth (Menezes et al., 2018) have provided insights into plastome evolution in the order Malpighiales, reporting rearrangements that are unique to Passifloraceae (Rabah et al., 2019; Shrestha et al., 2019), identifying regions of high sequence divergence, and helping resolve the phylogeny of the group.

Within the morphologically and ecologically diverse group Malpighiales, the family Podostemaceae stands out for its unusual habit (Xi et al., 2012). Riverweeds (as members of this family are also called) are notable for living attached to rocks in fast-flowing water habitats such as river rapids and waterfalls, with flowers that project above the water surface and fruits that develop and shed seeds only in the dry season when the water level is low (van Royen, 1951; Philbrick and Novelo, 1995; Rutishauser, 1995; Rutishauser, 1997; Philbrick and Novelo, 1998). Much remains to be explored in Podostemaceae despite a number of morphological (van Royen, 1951; Novelo and Philbrick, 1997; Rutishauser et al., 1999; Jäger-Zürn, 2011), developmental (Rutishauser, 1995; Rutishauser, 1997; Jäger-Zürn, 2005, Jäger-Zürn, 2007), and karyological (Oropeza et al., 1998; Oropeza et al., 2002) studies followed by phylogenetic and biogeographical investigations (Kita and Kato, 2001; Ruhfel et al., 2011; Tippery et al., 2011; Koi et al., 2012; Ruhfel et al., 2016).

The extreme conditions experienced by the Podostemaceae have resulted in highly modified vegetative and reproductive morphologies (Eckardt and Baum, 2010). Such forms constitute a taxonomical challenge because the high degree of modification of vegetative and reproductive structures results in a small number of morphological traits that are informative, making the study of the biology and evolution of this group difficult. Given this scenario, genomic data surface as the tool to gain better insight into the evolution of this notable group of plants.

In this study, we present the fully annotated plastid genomes of 5 species of Podostemaceae: *Apinagia riedelii* Tul., *Marathrum capillaceum* (Pulle) P. Royen, *Marathrum utile* Tul., *Monostylis capillacea* Tul., and *Tristicha trifaria* (Bory ex Willd.) Spreng. We analyzed our data in a comparative framework within Malpighiales to detect rearrangements and structural characteristics of the plastome of this distinctive family, taking advantage of the data already available in the order. A phylogenetic tree was inferred with whole-plastid data to test relationships and examine sequence divergence and amount of change within the family and order. Our investigation constitutes the first report of a complete nucleotide sequence and structure of the plastid genome in the Podostemaceae.

## Materials and Methods

### Taxon Sampling, DNA Extraction, and Sequencing

Samples of *A. riedelii*, *M. capillaceum*, *M. utile*, *M. capillacea*, and *T. trifaria* were collected in South America and Africa. Information on collection localities and voucher specimens is shown in **Table 1**. Together, these samples represent 2 of 3 subfamilies within Podostemaceae (Podostemoideae and Tristichoideae). Subfamily Tristichoideae is sister to a clade comprising the Podostemoideae and the monotypic Weddellinoideae (Kita and Kato, 2001). Therefore, any patterns shared between Tristichoideae and Podostemoideae would most likely be synapomorphies of the Podostemaceae. All species included have a distribution restricted to the Neotropics except for the pantropical *T. trifaria*.

**Table 1:** Provenance, voucher information, and/or GenBank accession numbers of the species in Malpighiales whose plastomes were included in this study.

Species	Family	Voucher (Herbarium)	GenBank accession no.	Collection locality
*Hirtella racemosa*	Chrysobalanaceae	—	NC_024060	
*Garcinia mangostana*	Clusiaceae		NC_036341	
*Byrsonima crassifolia*	Euphorbiaceae	—	NC_037192	
*Passiflora edulis*	Passifloraceae	—	NC_034285	
Apinagia riedelii*	Podostemaceae	C.P. Bove 2513 (R)	MN165812	Brazil, South America
Marathrum utile*	Podostemaceae	AMB 497 (ANDES)	MN165814	Colombia, South America
Marathrum capillaceum*	Podostemaceae	C.P. Bove 2493 (R)	MN165813	Brazil, South America
Monostylis capillacea*	Podostemaceae	C.P. Bove 2524 (R)	MN165815	Brazil, South America
Tristicha trifaria*	Podostemaceae	A. Mesterhazy MLI 128(Z)	MN165816	Mali, Africa
*Salix purpurea*	Salicaceae	—	NC_026722	
*Viola seoulensis*	Violaceae	—	NC_026986	

Voucher number and collection locality are provided only for those species whose genome was generated in this study (*).

Total genomic DNA was extracted from silica-dried leaf tissue using a modified CTAB protocol and purified by isopropanol precipitation, or *via* silica columns (Epoch Life Science, Missouri City, TX, USA) from the aqueous supernatant after chloroform/isoamyl alcohol purification (Neubig et al., 2014). DNA was run on a 1% agarose gel to assess DNA quality, and concentration was measured with a Qubit fluorometer using the dsDNA BR Assay Kit (Thermo Fisher Scientific, Waltham, MA, USA). A volume of 90 μL of total DNA of *M. utile* was used to prepare a library with an average fragment size of 500 bp, using the Kapa Biosystems Hyper prep kit at the QB3 Vincent J. Coates Genomics Sequencing Laboratory at UC Berkeley. Whole-genome shotgun sequencing was also performed at the QB3 Sequencing Laboratory, with 150 bp paired-end reads on 1 lane of an Illumina HiSeq4000. For the remaining species, a volume of 50 μL of 50 ng/μL total DNA was used to prepare libraries with average fragment size of 500 bp by Rapid Genomics LLC (Gainesville, FL, USA). Whole-genome sequencing of 150 bp paired-end reads was performed at the same facility by multiplexing samples in 1 lane of an Illumina HiSeqX.

### Plastome Assembly and Annotations

Read quality of paired-end Illumina reads was assessed in FastQC (https://www.bioinformatics.babraham.ac.uk/projects/fastqc/), and adapter sequences were removed using Trimmomatic (Bolger et al., 2014). The pipeline GetOrganelle (Jin et al., 2018) was used to select trimmed reads that corresponded to the plastid using the plastome of *Garcinia mangostana* L. (Clusiaceae) as a reference. The pipeline was also used to assemble the filtered reads. The annotations of the plastomes of *G. mangostana*, *Manihot esculenta* Crantz, and *Salix purpurea* L. (see **Table 1** for GenBank accession numbers) were transferred to the final circular plastid consensus sequences of *A. riedelii*, *M. utile*, *M. capillaceum*, *M. capillacea*, and *T. trifaria* with the tool “Annotate from source” in Geneious 9.1.8. (Biomatters Ltd., Auckland, New Zealand). Annotations were manually inspected, and tRNAs were further checked with tRNAscan-SE v2.0 as implemented in GeSeq (Tillich et al., 2017). GC content and boundaries between the IRa IRb, LSC, and SSC regions were determined in Geneious. The diagrams for the circular genomes were obtained with the program OGDRAW (Greiner et al., 2019).

In addition, a second approach to plastome assembly was conducted for *M. utile* to confirm the output of GetOrganelle. In this second assembly method, plastid filtered reads from GetOrganelle were imported in Geneious 9.1.8. The BBDuk tool was used to trim low-quality bases (Q20) and discard short reads (<10 bp). Reads were further normalized and error corrected using the tool BBNorm with target coverage level 30. A total of 225,896 filtered reads were assembled *de novo* using the Medium sensitivity/Fast option in the Geneious Prime *de novo* assembler. The options “Don’t merge variants” and “Produce scaffolds” were left unchecked.

In order to obtain a draft circular plastome, the consensus sequence of the largest contig (112,008 bp with 41.9X mean coverage) was generated. The Geneious Prime plugin “Find Repeats” was used in order to find the IRs. The *de novo* assembly of short reads in Geneious does not allow a full assembly of both IRs. Instead, it generates a consensus sequence with 1 full IR and the truncated ends of the second IR. For this reason, the latter were trimmed, and the single instance of the full IR was extracted. This extracted IR was reversed complement and concatenated with the previously trimmed consensus sequence of the largest contig. The generated draft genome was used as a reference to map the trimmed paired reads without normalization. This map-to-reference assembly was used for single nucleotide polimorphism (SNP) variant calling and to generate a final full circular plastid consensus sequence.

### Plastome of Podostemaceae in a Comparative Framework

To detect differences in the plastomes of the selected species of Podostemaceae with respect to other Malpighiales, we compared the assembled plastid genomes with six species representing six plant families in the order Malpighiales. The families included for comparison represent all the three major clades in Malpighiales (Xi et al., 2012). Accession numbers for the species included in this comparative analysis are listed in **Table 1**. Visual inspection of rearrangements was performed using progressive Mauve v.2.4.0 with default “seed families” and default values for all other parameters (Darling, 2004). As Mauve cannot handle duplicated regions, one of the IRs of each genome was manually removed following Firetti et al. (2017). The boundaries between the IRa IRb, LSC, and SSC regions in all species were inspected in Geneious using the fully assembled plastids.

We used the software mVista in Shuffle-LAGAN mode to explore variation in gene content within Malpighiales. *Garcinia mangostana* was used as reference in order to detect possible gene losses, gene variation, or gene conservation in Podostemaceae. Genes with <50% similarity were inspected directly in the annotated genomes of Podostemaceae to determine if they were intact, open reading frames. In a separate analysis, *A. riedelii* was used as reference to determine the level of similarity across the whole-plastome sequence in Malpighiales with respect to Podostemaceae.

In order to test relationships and examine sequence divergence and amount of change within both Malpighiales and Podostemaceae, a phylogenetic tree was inferred using the plastid genomes of all studied species. *Averrhoa carambola* L. (Oxalidaceae) was used as an outgroup to root the tree. To generate the alignment, in each species the IRb regions were deleted to remove duplicated genes; protein-coding regions, tRNAs, rRNAs, and noncoding regions were extracted, and all genes located on the reverse strand were reversed complemented. The extracted regions were aligned with MAFFT v7.309 in Geneious and then concatenated. The final alignment was 134,969 bp long. The software PartitionFinder2 (Lanfear et al., 2016) was used to select the best partitioning scheme, using a greedy search (Lanfear et al., 2012) in RAxML (Stamatakis, 2014). In the analysis, the three codon positions for each protein-coding region and each tRNA and rRNA were considered separately. Noncoding regions were analyzed together. Maximum likelihood phylogenetic inference was performed using RAxML v8.2 (Stamatakis, 2014), with the “rapid bootstrap analysis and search for best-scoring ML tree option” and 10,000 bootstrap replicates. Per-partition branch lengths were estimated independently.

## Results

### Genome Content and Structure in Podostemaceae

After sequencing, trimming, and selecting reads corresponding only to the plastids in GetOrganelle, 1,581,656 paired reads were recovered for *A. riedelii*, 1,443,458 for *M. utile*, 225,344 for *M. capillaceum*, 1,087,996 for *M. capillacea*, and 313,332 for *T. trifaria*. The largest plastome was that of *A. riedelii* with a length of 134,912 bp (1177.6X coverage), followed by *M. capillaceum* with 134,374 bp (190.8X coverage), *M. capillacea* with 133,944 bp (736.3X coverage), *M. utile* with 131,951 bp (1264.2X coverage), and *T. trifaria* with 130,285 bp (217.6X coverage). Assembly of the plastome of *M. utile* using Geneious 9.1.8 yielded the same sequence as with GetOrganelle, but mean coverage was lower (514.9X vs. 1264.2X).

**Figure 1:**
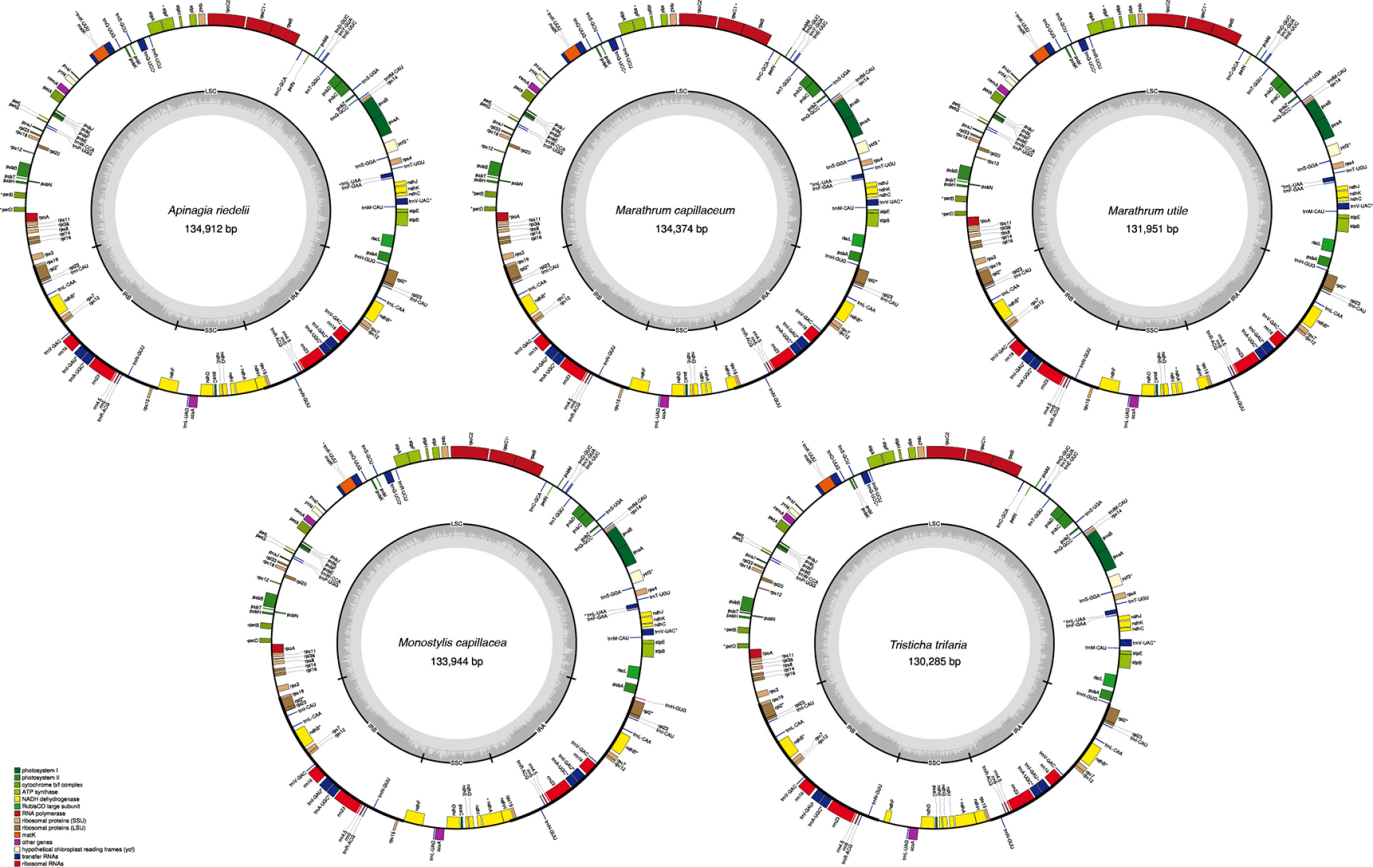
Plastid genomes of the 5 species of Podostemaceae included in this study. Only functional genes are drawn, and GC content graphs are included as dark gray bars toward the center of each diagram. Intron-containing genes are marked with (*).

All 5 full plastome assemblies in Podostemaceae showed the typical quadripartite structure characteristic of the plastids (see **Figure 1**). GC content in the IRs is higher than in other regions of the plastid, possibly due to the presence of tRNA genes, as suggested in Dipsacales (Fan et al., 2018). In the 5 species, the 2 IRs span 29.7% to 31.4% of the plastome (**Table 2**).

**Table 2:** Structural information of the plastid genomes of Podostemaceae, Clusiaceae, Malpighiaceae, Chrysobalanaceae, Violaceae, Passifloraceae, and Salicaceae. The percentages of the total size of the genome that corresponds to each region are included.

Species	Family	Plastome genome size (bp)	IRs length (bp)	SSC length (bp)	LSC length (bp)
*Apinagia riedelii*	Podostemaceae	134,912	21,049 × 2 (∼30.1%)	12,437 (∼8.9%)	85,377 (∼61%)
*Monostylis capillacea*	Podostemaceae	133,944	21,026 × 2 (∼31.4)	12,395 (∼9.3%)	79,497 (∼59.4%)
*Marathrum utile*	Podostemaceae	131,951	19,945 × 2 (∼30.2%)	12,283 (∼9.3%)	79,778 (∼60.5%)
*Marathrum capillaceum*	Podostemaceae	134,374	21,041 × 2 (∼31.3)	12,302 (∼9.2%)	79,990 (∼59.5%)
*Tristicha trifaria*	Podostemaceae	130,285	19,349 × 2 (∼29.7)	12,662 (∼9.7%)	78,925 (∼60.6%)
*Garcinia mangostana*	Clusiaceae	158,179	27,009 × 2 (∼34.1%)	17,704 (∼11.2%)	86,457 (∼54.7%)
*Byrsonima crassifolia*	Malpighiaceae	160,212	26,975 × 2 (∼33.7%)	17,814 (∼11.1%)	88,448 (∼55.2%)
*Hirtella racemosa*	Chrysobalanaceae	162,891	26,866 × 2 (∼33%)	19,915 (∼12.2%)	89,244 (∼54.8%)
*Viola seoulensis*	Violaceae	156,507	26,404 × 2 (∼33.7%)	18,008 (∼11.5%)	85,691 (∼54.8%)
*Passiflora edulis*	Passifloraceae	151,406	26,152 × 2 (∼34.5%)	13,378 (∼8.8%)	85,724 (∼56.6%)
*Salix purpurea*	Salicaceae	155,590	27,459 × 2 (∼35.3%)	16,220 (∼10.4%)	84,452 (∼54.3%)

Gene content was the same across the Podostemaceae species studied, with each genome including 71 protein coding genes, 30 tRNAs, and 4 rRNAs for a total of 105 genes, 13 of which contain 1 intron and 1 (*trnK-UUU*), which contains 2 introns. Of the total number of genes, 77 (∼73.33) occur in the LSC, 10 (∼9.52%) in the SSC, and 18 (∼17.14%) in the IRs. With regard to protein coding genes, 55 (∼77.46%) are included in the LSC, 9 (∼12.68%) in the SSC, and 7 (∼9.86%) in the IRs. Most tRNAs exist in the LSC region with 28 (∼73.33%) tRNAs, followed by 7 (∼23.33%) in the IRs, and only 1 (∼3.33%) in the SSC region. All rRNAs were found in the IRs. A full account of gene content for the Podostemaceae species is listed in **Table 3**.

**Table 3:** Gene content in all Podostemaceae species included in this study.

Gene function	Gene group	Gene name
Self-replication	Ribosomal RNA genes	***rrn 4.5, rrn5, rrn16, rrn23***
	Transfer RNA genes	trnA-UGC*, *trnC-GCA*, *trnD-GUC*, *trnE-UUC*, *trnF-GAA*, *trnfM-CAU*, *trnG-GCC*, trnG-UCC*, *trnH-GUG*, ***trnI-CAU***, trnI-GAU*, trnK-UUU*, ***trnL-CAA***, trnL-UAA*, *trnL-UAG*, *trnM-CAU*, ***trnN-GUU***, *trnP-UGG*, *trnQ-UUG*, ***trnR-ACG***, *trnR-UCU*, *trnS-GCU*, *trnS-GGA*, *trnS-UGA*, *trnT-GGU*,* trnT-UGU*, ***trnV-GAC***, trnV-UAC*, *trnW-CCA*, *trnY-GUA*
	Small subunit of ribosome	*rps2*, *rps3*, *rps4*, ***rps7***, *rps8*,* rps11*, ***rps12***, *rps14*, ***rps15***, *rps18*, ***rps19***
	Large subunit of ribosome	rpl2*, *rpl14*, *rpl16*, *rpl20*, *rpl33*, *rpl36*
	RNA polymerase subunits	*rpoA*, *rpoB*, rpoC1*, *rpoC2*
Photosynthesis	Subunits of NADH dehydrogenase	ndhA*, ndhB*, *ndhC*, *ndhD*, *ndhE*, *ndhF*, *ndhG*, *ndhH*, *ndhI*, *ndhJ*, *ndhK*
	Subunits of photosystem I	*psaA*, *psaB*, *psaC*, *psaI*, *psaJ*, ycf3*
	Subunits of photosystem II	*psbA*, *psbB*, *psbC*, *psbD*, *psbE*,* psbF*, *psbH*, *psbI*, *psbJ*, *psbK*, *psbL*, *psbM*, *psbN*, *psbT*, *psbZ*
	Subunits of cytochrome b/f complex	*petA*, petB*, petD*, *oetG*, *petL*,* petN*
	Subunits of ATP synthase	*atpA*, *atpB*, *atpE*, atpF*, *atpH, atpI*
	Large subunit of Rubisco	*rbcL*
Other	Maturase	*matK*
	Envelope membrane protein	*cemA*
	C-type cytochrome synthesis	*ccsA*
	ORFs	*ycf4*

Genes in bold correspond to genes that are located in the IRs and hence are duplicated. Genes that contain introns are marked with asterisk (*).

### Plastome of Podostemaceae in a Comparative Framework Within Malpighiales

**Figure 2:**
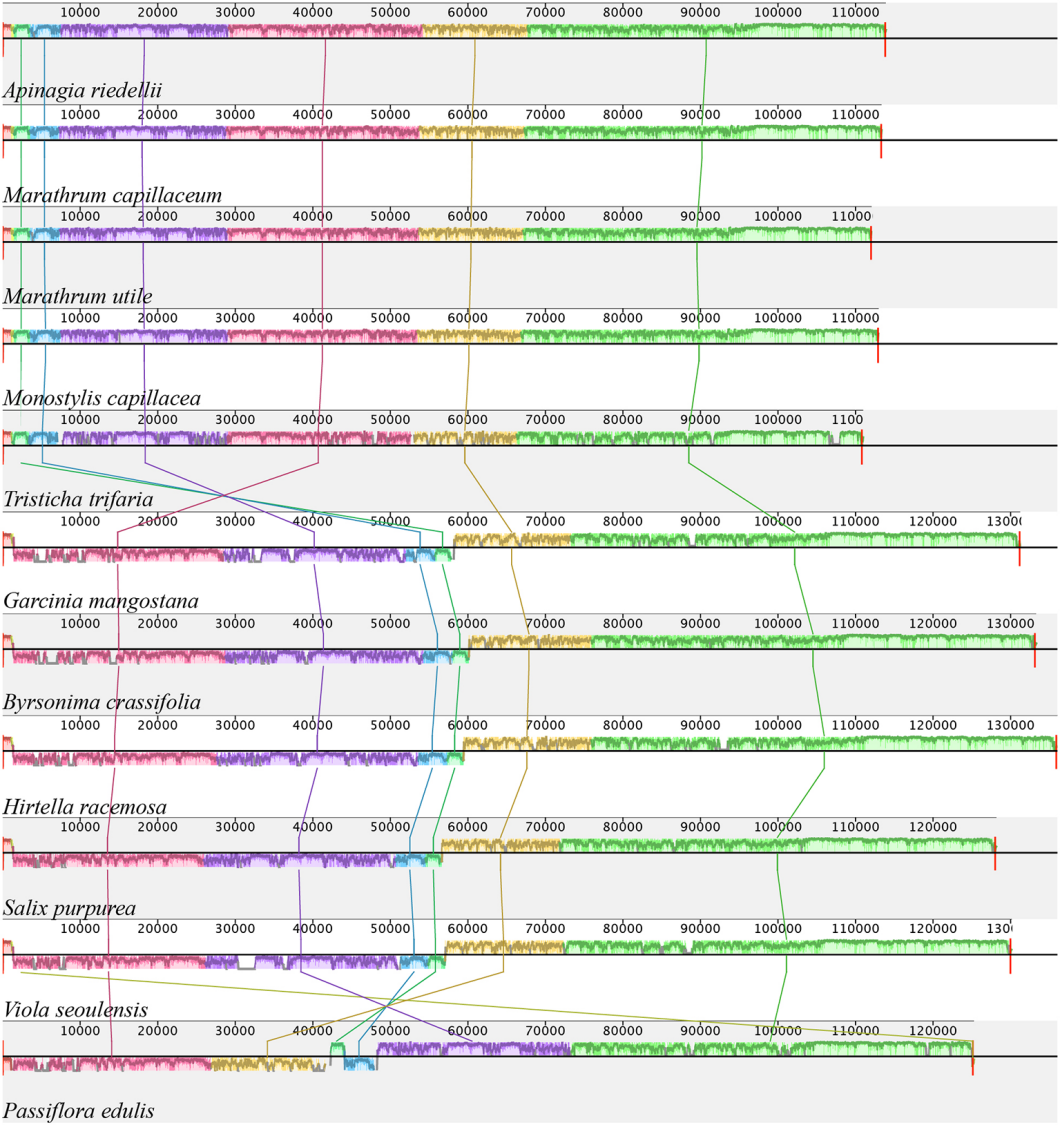
Alignment resulted from Mauve showing a large inversion shared by all Podostemaceae. Color bars indicate syntenic blocks, and connecting lines indicate correspondence of blocks across genomes.

Information on plastid genome size and size of the IRa, IRb, LSS, and SSC regions in all species shows that the Podostemaceae possess the smallest genome of the species included in this study (**Table 2**). This reduction is relatively uniform across the IRs, LSS, and SSC, as the proportions of each region in the plastid remain fairly similar in Malpighiales. However, in Podostemaceae, the LSC region did not shrink as much as the SSC and IRs regions, occupying a slightly larger percentage of the plastid in Podostemaceae (**Table 2**). Inspection of the plastomes of Podostemaceae and selected members of the Malpighiales with Mauve shows a large inversion of ∼49,000 bp in the LSC region. The inversion is located between the genes *rbcL* and *trnK*. This rearrangement is unique in Podostemaceae with respect to the other Malpighiales species inspected (**Figure 2**). Other rearrangements are seen in *P. edulis* as previously reported (Cauz-Santos et al., 2017; Shrestha et al., 2019).

**Figure 3:**
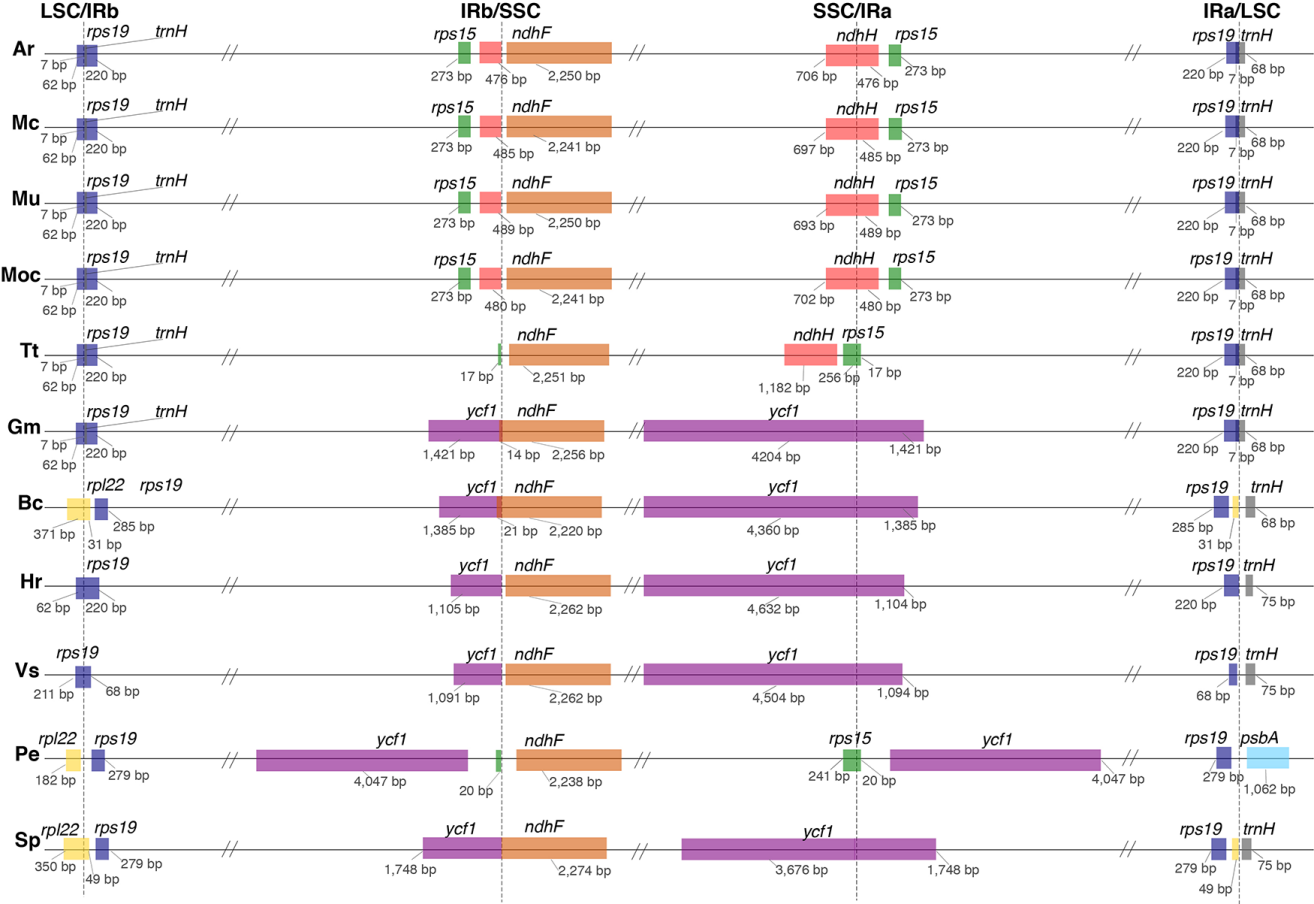
Comparison of border positions of the 4 plastid regions (LSC, IRb, SSC, IRa) among plastomes of Ar, *Apinagia riedelii;* Mc, *Marathrum capillaceum;* Mu, *Marathrum utile*; MoC, *Monostylis capillacea*; Tt, *Tristicha trifaria;* Gm, *Garcinia mangostana;* Bc, *Byrsonima crassifolia;* Hr, *Hirtella racemosa;* Vs, *Viola seoulensis;* Pe, *Passiflora edulis;* Sp, *Salix purpurea*. Functional genes and truncated fragments are shown with the same color. The sizes of fragments in genes that are located in a boundary are shown.

A comparison of border positions of the four plastid regions in the full organelle sequences across the 11 species studied is shown in **Figure 3**. The LSC/IRb border is located within the *rps19* gene, creating a 220-bp truncated copy (pseudogene) in the IRa in all the Podostemaceae species studied, as well as in *G. mangostana* and *Hirtella racemosa* Lam. In *Viola seoulensis* Nakai, this duplicated fragment is only 68 bp, in line with previous work (Menezes et al., 2018). Variations in the length of the IRb in *B. crassifolia*, *S. purpurea*, and *P. edulis* caused the LSC/IRb border to fall within the *rpl22* gene in the former two species, and between *rpl22* and *rps19* in *P. edulis*. This created a pseudogene in the IRa of both *B. crassifolia* and *S. purpurea*. In Podostemaceae and in *G. mangostana*, the boundaries of *trnH* and the truncated copy of *rps19* overlap by 7 bp in the IRa. In all species except in *P. edulis*, *trnH-GUG* is the first gene in the LSC region. This exception has been proposed to be caused by a small inversion at the beginning of the LSC region containing the *psbA* and *trnH-GUG* genes (Cauz-Santos et al., 2017). The SSC/IRa is located within the *ndhH* gene in *A. riedelii*, *M. capillaceum*, *M. capillacea*, and *M. utile*, creating a pseudogene in the IRb. This border is shifted to the *rps15* gene in *T. trifaria* and *P. edulis*, where a small fragment of this gene (< 20 bp long) spans the IRa and is duplicated in the IRb. In the remaining species, the SSC/IRa border falls in the *ycf1* gene, which is located downstream of the *ndhH* and *rps15*. As a consequence, a *ycf1* pseudogene is produced in the IRb. This gene is reduced to a pseudogene in Podostemaceae.

**Figure 4:**
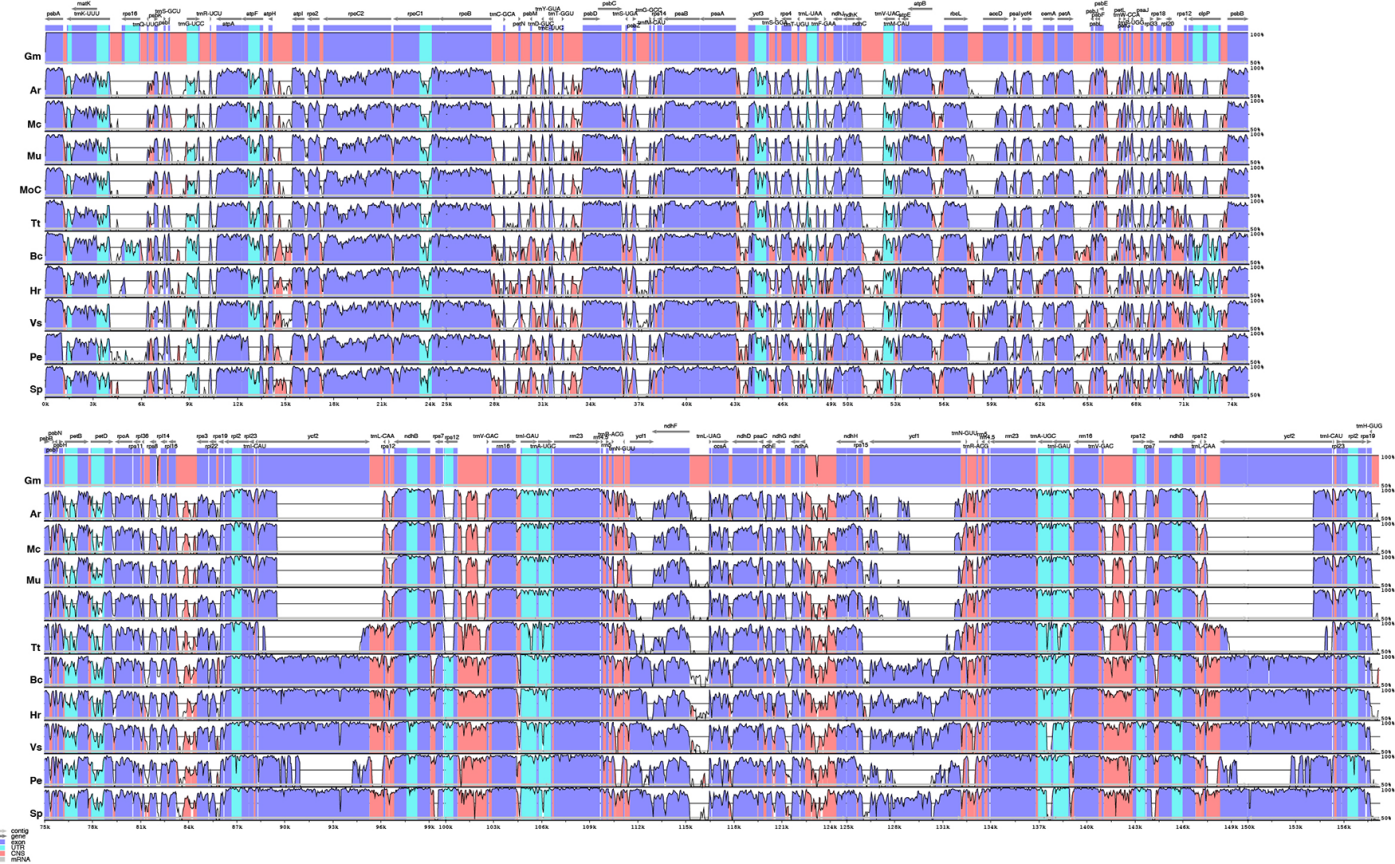
Comparison of percentage identity of plastomes in mVista using *Garcinia mangostana* (Gm) as reference. Ar, *Apinagia riedelii*; Mc, *Marathrum capillaceum*; Mu, *Marathrum utile*; Moc, *Monostylis capillacea*; Tt, *Tristicha trifaria*; Bc, *Byrsonima crassifolia*; Hr, *Hirtella racemosa*; Vs, *Viola seoulensis*; Pe, *Passiflora edulis*; Sp, *Salix purpurea*. The vertical axis corresponds to the percentage identity (50%–100%), while the horizontal axis shows the position of each region within the locus. Arrows indicate the transcription of annotated genes in the reference genome. Genome regions are color coded.

An alignment of 11 species in six families with *G. mangostana* used as reference is shown in **Figure 4**. In this alignment, the large inversion previously identified was reinverted in order to enhance visualization and allow gene content comparison. We found that species in Podostemaceae share the loss of the *rps16* gene with most other Malpighiales, except for *B. crassifolia* (Malpighiaceae), where the gene is present. Similarly, the Podostemaceae are like other Malpighiales in the retention of the *atpF* Group II intron, which is absent only in *P. edulis*. On the contrary, the gene for the subunit of acetyl-Co-A-carboxylase (*accD*) is highly divergenet in the Podostemaceae and not in frame in M. capillacea and in M. capillaceum. The large subunit of ribosome protein (rpl23), and the chloroplast open reading frames *ycf1* and *ycf2* are reduced to pseudogenes only in Podostemaceae and in *P. edulis* (Cauz-Santos et al., 2017) (**Figure 3**).

**Figure 5:**
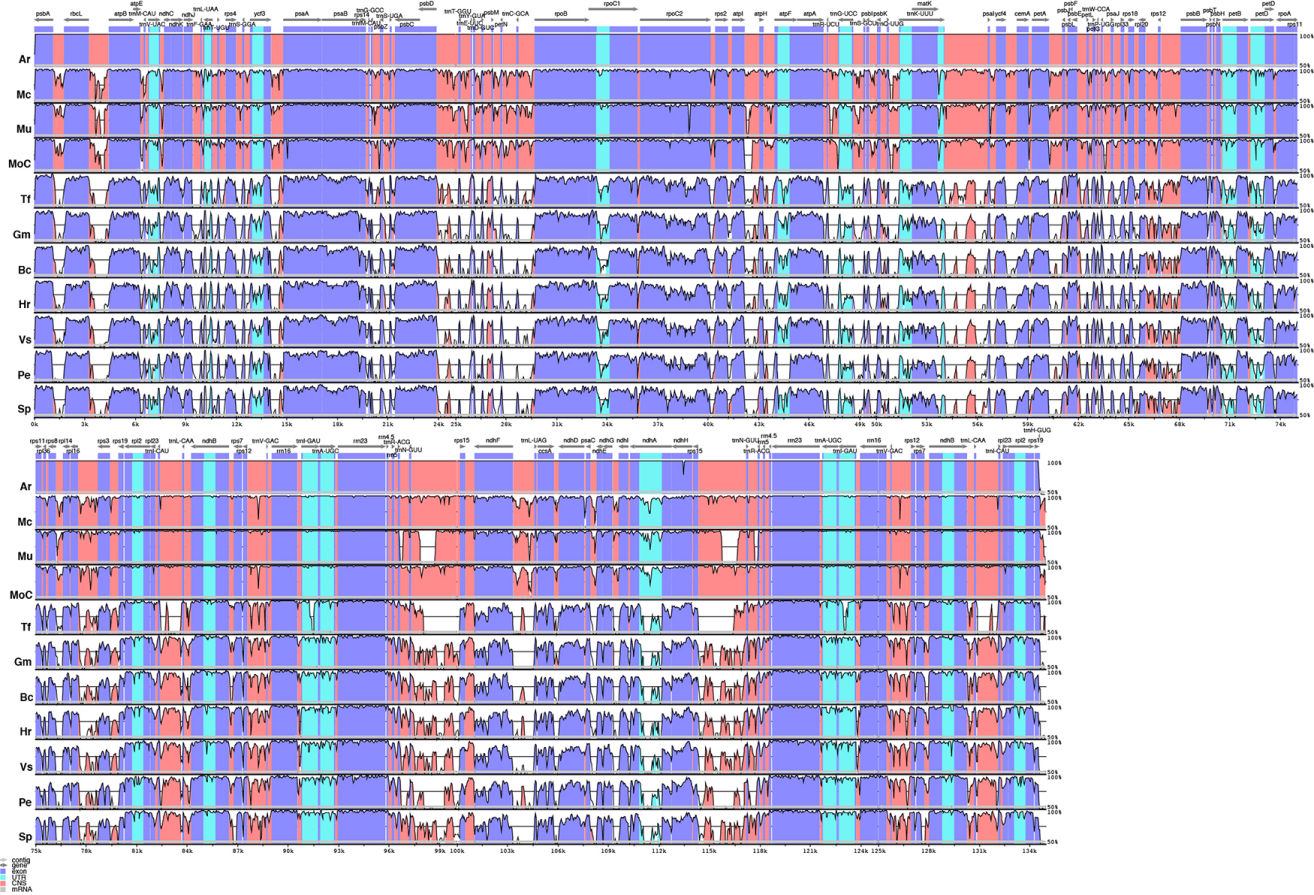
Comparison of percentage identity of plastomes in mVista using *Apinagia riedelii* (Ar) as reference. Mc, *Marathrum capillaceum*; Mu, *Marathrum utile*; Moc, *Monostylis capillacea*; Tt, *Tristicha trifaria*; Gm, *Garcinia mangostana*; Bc, *Byrsonima crassifolia*; Hr, *Hirtella racemosa*; Vs, *Viola seoulensis*; Pe, *Passiflora edulis*; Sp, *Salix purpurea*. The vertical axis corresponds to the percentage identity (50%–100%), while the horizontal axis shows the position of each region within the locus. Arrows indicate the transcription of annotated genes in the reference genome. Genome regions are color coded.

The analysis performed in mVista using *A. riedelii* as reference is shown in **Figure 5**. *Apinagia riedelii*, *M. capillaceum*, *M. utile*, and *M. capillacea*, all members of the Podostemoideae, show high similarity across their plastome. In fact, the percentage similarity supports that all four species belonging to this subfamily are more similar to each other than any of them are to *Tristicha*, in the subfamily Tristichoideae. As expected, similarity is higher in coding regions than in intergenic sequences.

**Figure 6:**
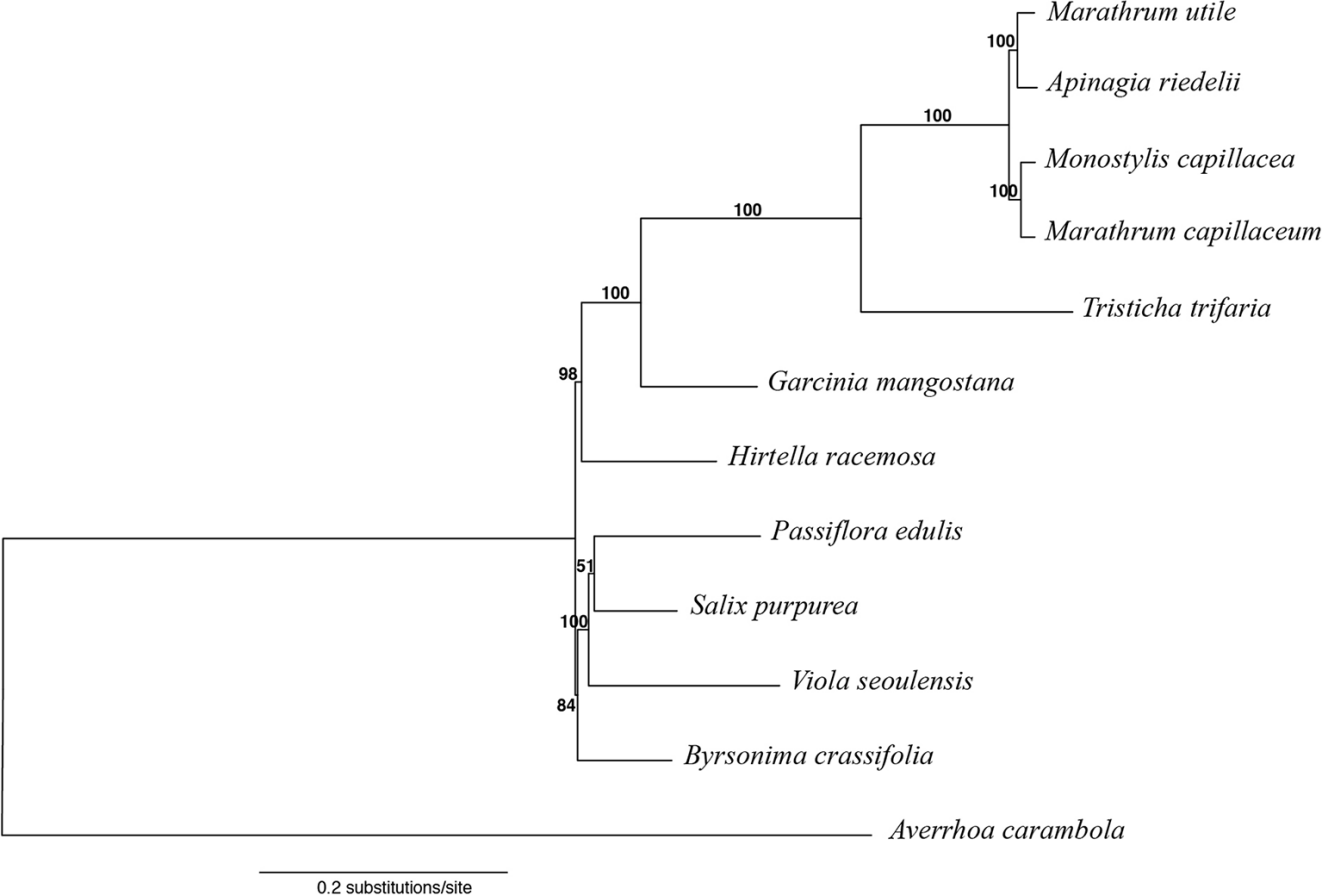
Maximum likelihood tree obtained with RAxML, using *Averrhoa carambola *as outgroup for rooting. Bootstrap support is shown above branches.

Phylogenetic analysis was conducted using an optimal scheme with 53 partitions as resulted from PartitionFinder2. Information on partitions and substitution models is included in the **Supplementary Material**. Among the Podostemaceae, the Podostemoideae are supported as monophyletic and sister to *T. trifaria* (**Figure 6**). The phylogeny also shows that the branches leading to taxa in the Podostemaceae from the common ancestor of Malpighiales are much longer than the branches leading to other taxa within the order. *Garcinia mangostana* (Clusiaceae) is supported as sister to Podostemaceae (100% bootstrap), in line with previous work, but this clade was found as sister to *H. racemosa* (Chrysobalanaceae), contrary to previous work (Xi et al., 2012; Menezes et al., 2018) where Chrysobalanaceae is found as more closely related to Malpighiaceae. *Salix purpurea*, *P. edulis*, and *V. seoulensis* are supported as a clade (100% bootstrap), and the relationships among them are in agreement with Xi et al., 2012. However, *B. crassifolia* (Malpighiaceae) is reconstructed as sister to this clade (85% bootstrap), and as mentioned above, this contradicts previous published work (Xi et al., 2012; Menezes et al., 2018).

## Discussion

The 130,218- to 134,912-bp size range of the plastome reported in this study for Podostemaceae species falls within the average size of angiosperm plastomes (Sugiura, 1992). However, it is notable that the full plastid genomes generated here for the family are among the smallest reported so far in Malpighiales (Shrestha et al., 2019; https://www.ncbi.nlm.nih.gov/genome).

It has been proposed that plastome size variation could be caused by variation in length of IR regions, gene loss, and intergenic region variation (Palmer et al., 1987; Wolfe et al., 1992; Wakasugi et al., 1994; Chumley et al., 2006; Xiao-Ming et al., 2017). We have reported here that the IRs in the Podostemaceae are ∼6 kb smaller than in the other Malpighiales used for comparison (**Table 2**), and we have also reported the loss of *rps16* and reduction to pseudogenes of *accD* (in some species of Podostemaceae), *ycf1*, and *ycf2*. However, the average size of the plastome of Podostemaceae is smaller than the other Malpighiales examined here by 16 to 28 kb, and this difference cannot be explained by a smaller length of the IRs and by gene losses alone. Intergenic region variation as well as intron loss also contribute to this difference in plastome size, considering that the number of introns reported for Podostemaceae is smaller than in *P. edulis* (Cauz-Santos et al., 2017) and that intergenic regions are the most variable in our comparative study (**Figure 4**). Indeed, when calculating the total length of intergenic regions in Podostemaceae and of the other species in Malpighiales analyzed here, the Podostemaceae are shorter by ∼5.5 kp on average. This implies that all three processes responsible for genome size variation mentioned above are responsible for the reduction in size of the plastomes in Podostemaceae.

The large rearrangement in the LSC region appears to be a synapomorphy of Podostemaceae, but this observation should be confirmed in more species in the family before this trait is considered to be of any systematic relevance. Other structural rearrangements have been reported in Malpighiales such as the 3 inversions in the LSC region in *P. edulis* (Cauz-Santos et al., 2017), high rates of rearrangements in *Passiflora* (Rabah et al., 2019; Shrestha et al., 2019), and a single small inversion in the LSC region of *Hevea brasiliensis* (Tangphatsornruang et al., 2011). We found no evidence of other structural rearrangements within Podostemaceae.

Evaluation of the boundaries of the 4 plastid regions across all species suggests that the locations of borders of the IRs in the Podostemoideae sampled are fairly conserved, but differ to a small degree in all 5 species studied. This is consistent with the IR boundaries being in a dynamic state in most angiosperms (Goulding et al., 1996). A change in length in the IRs of *T. trifaria*, which are slightly smaller than in Podostemoideae (**Table 2**), could be interpreted as either a contraction of the IRs in *T. trifaria* or an expansion of the regions in Podostemoideae. Either way, expansions and contractions of the IRs have occurred more than once in Malpighiales, creating pseudogenes (Cauz-Santos et al., 2017; Menezes et al., 2018; Shrestha et al., 2019). Podostemaceae are no exception to these variations in length, but as mentioned above, these do not seem to be the sole reason why Podostemaceae have one of the smallest plastomes in Malpighiales.

With regard to gene content, the retention of the *atpF* Group II intron is considered an ancestral condition in land plants with a single gain within the streptophytes, before the origin of land plants, followed by losses in charophytes (Daniell et al., 2008). This intron has also been found to be lost from the plastome of members of Euphorbiacceae, Phyllanthaceae, Elatinaceae, Lophopixidaceae, and Passifloraceae (Daniell et al., 2008). Podostemaceae is a lineage within Malpighiales that retains the ancestral state for presence of the *atpF* group II intron.

Targeted gene disruptions in tobacco have identified four plastid genes with essential functions beyond photosynthesis: *accD*, *clP*, *ycf1*, and *ycf2* (Drescher et al., 2000; Kuroda and Maliga, 2003; Kode et al., 2005; Kikuchi et al., 2013; Parker et al., 2014; Dong et al., 2015). Even though these four genes are retained in the plastid genomes of most angiosperms, including parasitic species that are chlorophyll-deficient (dePamphilis and Palmer, 1990; Funk et al., 2007; Jansen et al., 2007; Parker et al., 2014), there are multiple other parasitic, mycoheterotrophic plants, and taxa outside Malpighiales where these genes are missing from the plastids (Kim, 2004; Magee et al., 2010; Lei et al., 2016; Graham et al., 2017). As reported here, some of these genes may have been reduced to pseudogenes independently in Podostemaceae and in *Passiflora* (Shrestha et al., 2019).

The pseudogenization or loss of genes from the plastids has been reported to be a consequence of it being transferred to the nuclear genome (Jansen et al., 2011; Cauz-Santos et al., 2017). This event of plastid gene transfer remains to be examined in Podostemaceae. The *rps16* gene is considered to be present in the common ancestor of land plants (Daniell et al., 2016) and is found in the plastomes of most angiosperms (Ueda et al., 2008). However, it has been repeatedly reported as lost in Malpighiales (Asif et al., 2010; Daniell et al., 2008; Jansen et al., 2007; Steane, 2005), including our findings of it being missing in Podostemaceae and in other angiosperms (Keller et al., 2017). The multiple losses of *rps16* from the plastids have been explained by the fact that the nuclear encoded *rps16* is dually targeted to the mitochondria and the plastids (Ueda et al., 2008; Keller et al., 2017). This has also been reported to be responsible for the pseudogenization of rpl23 (Bubunenko et al., 1994). Examination of the presence of this gene in the mitochondrial and nuclear DNA would be necessary to test if this explanation also applies to Podostemaceae.

The *ycf1* gene is one of the largest and most variable genes in the plastid genome of land plants, and as mentioned above, it has been proposed to be fundamental for plant function as a key component of the general protein import channel (Dong et al., 2015; Kikuchi et al., 2013). It is rarely missing from the plastome of autotrophic plant lineages, with the exception of Poaceae, some species of *Passiflora*, *Vaccinium macrocarpon*, and some species of *Erodium* (de Vries et al., 2015). However, this gene is more commonly lost from the organellar genome of parasitic, mycoheterotrophic, and carnivorous plant taxa such as *Orobanche purpurea*, species in Droseraceae, and a number of orchids (Guisinger et al., 2010; Parker et al., 2014; Graham et al., 2017; Nevill et al., 2019). Our finding that *ycf1* is pseudogenized in Podostemaceae adds this group to one of the unique autotrophic lineages in angiosperms where this is known to have occurred. However, the mechanisms that compensate for this loss and the implications of it remain to be studied.

The high similarity across the plastome in the subfamily Podostemoideae (**Figure 5**), which are more similar to each other than they are to *T. trifaria*, is explained by the fact that the members of this subfamily share a more recent common ancestor (**Figure 6**). The short branches within Podostemoideae indicate that fewer changes have accumulated since the species diverged, possibly as a consequence of recent speciation events with little subsequent sequence evolution (Soltis et al., 2019). Additionally, the fact that the branches leading to taxa in the Podostemaceae from their common ancestor in Malpighiales are much longer than the branches leading to other taxa within the order is an indicator of faster rates of evolution in the plastome of riverweeds, giving support to previous suggestions (Ruhfel et al., 2016).

Long branches depicting accelerated rates of evolution have been reported in parasitic plants, where multiple changes in the chloroplast respond to a switch from an autotrophic to a heterotrophic metabolism, causing a reduced function of the genome (Young and dePamphilis, 2005; Stefanovic et al., 2007; Lemaire et al., 2011; Givnish et al., 2018). However, the switch from autotrophy to heterotrophy has not occurred in the Podostemaceae. Instead, faster rates of evolution in Podostemaceae could be explained by their rapid life cycle and shorter generation times; most species of Podostemaceae are annual herbs because they depend on the water level to complete their life cycle, dying and shedding seeds in the dry season when the water level is low. This inverse correlation between evolutionary rate and generation time has been suggested for plants as well as for other organisms such as mammals (Bromham et al., 1996; Verdú, 2002; Smith and Donoghue, 2008). Interestingly, the same pattern of long branches observed in Podostemaceae has been found in the Hydrostachyaceae (Cornales) based on phylogenetic analysis using plastid data (Olmstead et al., 2000; Albach et al., 2001; Fan and Xiang, 2003), and the Hydrostachyaceae are the only angiosperm family that shares the unique habit of Podostemaceae (Jäger-Zürn, 1998; Qiu-Yun Xiang, 1999; Rutishauser et al., 2005). However, faster rates of evolution have also been correlated to other life history traits such as plant height, genome size, and age at first reproduction among others (Lehtonen and Lanfear, 2014; Bromham et al., 2015). Which factors are responsible for faster rates of evolution in Podostemaceae and whether they (it) has anything to do with the habit of Podostemaceae and Hydrostachyaceae, remain to be determined.

The phylogenetic relationships found here for the selected species of Malpighiales (**Figure 6**) are in line with previous work where Salicaceae and Passifloraceae are in a clade that shares a more recent common ancestor with Violaceae (and Goupiaceae), whereas Clusiaceae and Podostemaceae are together in a separate clade (Xi et al., 2012; Cai et al., 2019). The relationships within Podostemaceae also follow previous work that suggest that *Marathrum* is paraphyletic (Tippery et al., 2011; Philbrick et al., 2018), calling for a revision of the classification of the genus. Our results (**Figure 6**) also follow a recent study (Cai et al., 2019) in the placement of Chrysobalanaceae, using 5,113 orthology clusters to infer a phylogeny of Malpighiales. These results contradict previous works (Xi et al., 2012; Menezes et al., 2018) that have placed Chrysobalanaceae and Malpighiaceae as more closely related to one another than they are to any of the other families in the Malpighiales included here. The incongruence across data sets is in the deep nodes within the order, reinstating the difficulty in reconstructing deep nodes in Malpighiales (Wurdack and Davis, 2009).

## Conclusions

In this study, we assembled five full plastid genomes of species in Podostemaceae and analyzed them in a comparative framework within Malpighiales. We detected an important inversion in the LSC region that could be of systematic relevance as a synapomorphy of the group and also described slight variations in the length of the IRs in all the species included in the study. The plastomes of the family are among the smallest reported to date in the order Malpighiales, and we suggest that this small size is a result of a combination of variation in length of IR regions, gene loss, and intergenic region variation and intron loss. Gene content is the same within the Podostemaceae, and some of the gene loss and pseudogenization events reported are common in angiosperms (e.g., *rps16*, *rpl23*, and *accD*), whereas others are very rare (e.g., *ycf1* and *ycf2*). The mechanisms that compensate for these losses and the implications of their occurrence in Podostemaceae remain a subject of study. Our results suggest an accelerated rate of evolution for the group and reinstate the difficulty in the inferring relationship in deep nodes in Malpighiales. Ultimately, this study provides insights into the structure and evolution of plastomes in Podostemaceae and lays the foundations for phylogenomic studies in the family.

## Data Availability

Whole-plastid genome sequences are deposited in GenBank, and accession numbers are provided in **Table 1**. The records can be found in GenBank (https://www.ncbi.nlm.nih.gov/genbank/).

## Author Contributions

AB and RO designed the study and wrote the manuscript. AB analyzed the data and conducted field work in Colombia, collecting *Marathrum utile*. BR and CP contributed tissue samples, sequences, and comments. SM provided help with collecting permits and sampling logistics in the field in Colombia. CB contributed tissue samples of *A. riedelii*, *Marathrum capillaceum* and *Monostylis capillacea* from Brazil, as we all comments to the manuscript. AM provided tissue samples of *Tristica trifaria* from Africa.

## Funding

AB was supported by the Sargent award from the Biology department at the University of Washington, the Colciencias fellowship for Graduate studies (Doctorados en el Exterior-679), and the BSA and ASPT Research Grants for graduate students. This work used the Vincent J. Coates Genomics Sequencing Laboratory at UC Berkeley, supported by NIH S10 OD018174 Instrumentation Grant. BR was supported by a National Science Foundation Grant (DEB-1754329). Collection of some tissue samples was done with support of the National Science Foundation Grants (DEB-0444589 and DEB-1754199) and Connecticut State University-AAUP research grants to CP, and Conselho Nacional de Desenvolvimento Científico e Tecnológico (CNPq) grants PROTAX 562251/2010-3, REFLORA 563534/2010-9, and Productivity Grant (307870/2014-6) to CB. Open Access page charges covered in part by the Universidad de los Andes research results publication fund CI-001, and by the University of Washington and University of Michigan.

## Conflict of Interest Statement

The authors declare that the research was conducted in the absence of any commercial or financial relationships that could be construed as a potential conflict of interest.
