# Corrigendum: Plastid Genomes of Five Species of Riverweeds (Podostemaceae): Structural Organization and Comparative Analysis in Malpighiales

**DOI:** 10.3389/fpls.2020.598458

**Published:** 2020-11-09

**Authors:** Ana M. Bedoya, Bradley R. Ruhfel, C. Thomas Philbrick, Santiago Madriñán, Claudia P. Bove, Attila Mesterházy, Richard G. Olmstead

**Affiliations:** ^1^Department of Biology and Burke Museum, University of Washington, Seattle, WA, United States; ^2^University of Michigan Herbarium, University of Michigan, Ann Arbor, MI, United States; ^3^Department of Biological and Environmental Sciences, Western Connecticut State University, Danbury, CT, United States; ^4^Laboratorio de Botánica y Sistemática, Departamento de Ciencias Biológicas, Universidad de los Andes, Bogotá, Colombia; ^5^Departamento de Botânica, Museu Nacional, Universidade Federal do Rio de Janeiro, Rio de Janeiro, Brazil; ^6^Directorate of Hortobágy National Park, Debrecen, Hungary

**Keywords:** genome rearrangements, Malpighiales, phylogenomics, plastome, Podostemaceae

In the original article, there was an error. Throughout the paper it was stated that *ycf1, ycf2, accD, rpl22*, and *clpP* were pseudogenized in Podostemaceae. However, new inspection of our data revealed that *rpl22* and *clpP* are in frame and therefore, their functionality cannot be assessed based on sequence data alone. *accD* is highly divergent in Podostemaceae but is in frame in *Apinagia riedellii, Marathrum utile*, and *Tristicha trifaria*. The gene is not in frame in *Marathrum capillaceum* and in *Monostylis capillacea*. Therefore, we confirm that *accD* may be a pseudogene in two of the evaluated Podostemaceae species, but it may be functional in three of them. We also found that *rpl23*, reported as present in our study, is not in frame and that *rpl32*, not mentioned in our paper, is present and functional.

The authors apologize for this error and state that this does not change the scientific conclusions of the article in any way. The original article has been updated.

