# Supplementary material for: Plastid Genomes of Five Species of Riverweeds (Podostemaceae): Structural Organization and Comparative Analysis in Malpighiales
Source: Front Plant Sci. 2019 Aug 20;10:1035. doi: 10.3389/fpls.2019.01035 (PMC6710714; doi:10.3389/fpls.2019.01035)
Supplement: Supplementary file 1 [file Table_1.docx]

Supplementary Material

Table S1. Optimal scheme obtained with PartitionFinder2. Subset (partition) number, per-partition best-fit models and loci included in each partition are included.

| **Subset** | **Best Model** | **Partition names** |
| --- | --- | --- |
| 1 | GTR+I+G | psbB_pos1, psbD_pos1, PetG_pos1, psbN_pos1, atpH_pos1, psbC_pos2 |
| 2 | GTR+I+G | ndhD_pos2, ndhG_pos2, ndhE_pos2, petL_pos2, psbK_pos3, psbI_pos3, psbZ_pos2, PetG_pos2, petN_pos2 |
| 3 | GTR | PetG_pos3, psbK_pos1, psaI_pos3, psbF_pos3, psbE_pos3, psbM_pos3 |
| 4 | GTR+I+G | ndhC_pos1, rs12e1_pos2, trnH, petL_pos1, rps4_pos3, psaI_pos1, ycf4_pos2, psbZ_pos1, psaJ_pos1 |
| 5 | GTR+G | atpI_pos2, psaJ_pos2, ndhC_pos2, psaI_pos2, psbM_pos2 |
| 6 | GTR+I+G | psaJ_pos3, psaC_pos3, psbI_pos1, rpl14_pos3, rpl36_pos3, rps3_pos2, rps15_pos3, rps8_pos3, rpl16_pos3 |
| 7 | GTR+G | rps3_pos1, rps18_pos2, rps8_pos2, rpl33_pos1 |
| 8 | GTR+G | rpoA_pos1, rps8_pos1, rpoC2_pos1, rps3_pos3, rpl16_pos1, rps18_pos1, rpl33_pos2, psbN_pos3 |
| 9 | GTR+G | psbD_pos3, psaA_pos1, psbB_pos3, psaB_pos3, rpl33_pos3, psbC_pos1, rbcL_pos3 |
| 10 | GTR+G | rps14_pos3, atpH_pos3, ndhJ_pos3, rps4_pos1, atpI_pos3, psbH_pos3, rps18_pos3, cemA_pos2, atpF_pos3, rpoB_pos2, rps2_pos1, ndhC_pos3 |
| 11 | GTR+I+G | psbC_pos3, psaB_pos2, psbB_pos2, psaA_pos3, ndhJ_pos2, trnRUCU, rps12e2e3_pos2 |
| 12 | GTR+G | rpl20_pos1, rpl20_pos3, ccsA_pos1, cemA_pos3, ndhG_pos1, rs12e1_pos3, rpl20_pos2 |
| 13 | GTR+G | rs12e1_pos1, rpl36_pos1, rpl14_pos1, rps11_pos2 |
| 14 | GTR+I+G | trnFC, psbT_pos1, trnS, trnD, psbE_pos2, trnE, trnLUAG |
| 15 | GTR+G | ndhH_pos2, rpl14_pos2, psbT_pos2, rpoB_pos1, petA_pos2 |
| 16 | GTR+G | psbA_pos3, petL_pos3, psbT_pos3 |
| 17 | GTR+I+G | psbH_pos1, petN_pos1, psbM_pos1, psbE_pos1 |
| 18 | GTR+I+G | rbcL_pos2, psbH_pos2 |
| 19 | GTR+I+G | psbA_pos2, psbD_pos2, atpH_pos2, psbF_pos2, psbN_pos2 |
| 20 | GTR+G | psbK_pos2, petB_pos1, petD_pos2, petD_pos1 |
| 21 | GTR+I+G | ndhF_pos3, petB_pos2, ndhI_pos3, ndhK_pos3, ndhH_pos3, ndhD_pos3, rps11_pos1 |
| 22 | GTR+G | ndhF_pos1, petB_pos3 |
| 23 | GTR+G | trnG, petD_pos3 |
| 24 | GTR+G | rps19_pos3, rps19_pos1, rps19_pos2 |
| 25 | GTR+I+G | rpoC2_pos2, rpoA_pos2, rpoC1_pos1, ndhI_pos2 |
| 26 | GTR+G | rpoC2_pos3, atpA_pos3, rpoA_pos3, petA_pos3, ycf4_pos3 |
| 27 | GTR+I+G | rpl16_pos2, ndhK_pos2, ndhH_pos1, rps11_pos3 |
| 28 | GTR+I+G | rpl36_pos2, trnfM, trnC, trnQ |
| 29 | GTR+G | rpl2_pos2, rpl2_pos1 |
| 30 | GTR+G | rpl23_pos2, rpl23_pos3, rpl23_pos1, rpl2_pos3 |
| 31 | GTR+G | ndhB_pos2, ndhB_pos1, ndhB_pos3, trnI |
| 32 | GTR+G | rps7_pos3, rps7_pos1, rps7_pos2 |
| 33 | GTR+G | rps12e2e3_pos1, psbI_pos2, rps14_pos1, ndhE_pos1, rps12e2e3_pos3, rps2_pos3, trnS2, rps14_pos2 |
| 34 | GTR+I+G | ccsA_pos2, ndhF_pos2 |
| 35 | GTR+G | ndhA_pos1, ccsA_pos3 |
| 36 | GTR+I+G | cemA_pos1, rps15_pos1, rps15_pos2 |
| 37 | GTR+G | ndhA_pos2, ndhA_pos3 |
| 38 | GTR+G | rps2_pos2, petN_pos3, ndhI_pos1, ycf4_pos1, rps4_pos2, ndhK_pos1 |
| 39 | GTR+G | ndhE_pos3, psbZ_pos3, ndhG_pos3 |
| 40 | GTR+I+G | rrn45, psbF_pos1, trnT2, psaC_pos2, trnR, trnN, rrn5, psbA_pos1, trnT, trnV, trnL, psaA_pos2, trnG2, psaC_pos1, trnY |
| 41 | GTR+G | ndhD_pos1 |
| 42 | GTR+I+G | atpA_pos1, petA_pos1, rpoB_pos3, ndhJ_pos1, atpI_pos1 |
| 43 | GTR+I+G | atpA_pos2 |
| 44 | GTR+G | rpoC1_pos3, atpF_pos1 |
| 45 | GTR+G | ycf3_pos3, ycf3_pos1, atpF_pos2, trnV2, ycf3_pos2 |
| 46 | GTR+I+G | rpoC1_pos2 |
| 47 | GTR+I+G | trnS3, trnIGAU, psaB_pos1, trnA |
| 48 | GTR+I+G | rbcL_pos1 |
| 49 | GTR+I+G | trnK |
| 50 | GTR+I+G | rrn23, rrn16 |
| 51 | GTR+G | trnW, trnP |
| 52 | GTR+I+G | trnL2 |
| 53 | GTR+I+G | noncoding |
